# Relation between Overweight/Obesity and Self-Rated Health Among Adolescents in Germany. Do Socio-Economic Status and Type of School Have an Impact on That Relation?

**DOI:** 10.3390/ijerph120202262

**Published:** 2015-02-16

**Authors:** Laura Krause, Thomas Lampert

**Affiliations:** Department of Epidemiology and Health Monitoring, Robert Koch Institute, General-Pape-Straße 62-64, Berlin 12101, Germany; E-Mail: t.lampert@rki.de

**Keywords:** overweight and obesity, self-rated health (SRH), socio-economic status (SES), type of school, social inequalities, adolescence, KiGGS, health survey, Germany

## Abstract

This study investigates the relation between overweight/obesity and self-rated health (SRH), and whether this relation varies by social factors. Data was taken from the German Health Interview and Examination Survey for Children and Adolescents (KiGGS, baseline 2003‒2006). For the definition of overweight and obesity, body mass index was calculated based on standardized height and weight measurements. SRH of adolescents (n = 6813, 11‒17 years) was raised with the question: “How would you describe your health in general?” The response categories were “very good”, “good”, “fair”, “poor”, and “very poor”. We dichotomized these responses into: “very good/good” *vs.* “fair/poor/very poor”. Socio-economic status (SES) in the family of origin and adolescents’ school type were analyzed as modifying factors. Prevalence and age-adjusted odds ratios with 95% confidence intervals were calculated by binary logistic regression models. We found that overweight and obese boys and obese girls reported fair to very poor SRH more often than their normal weight peers, and that these differences were more apparent in early than late adolescence. In addition, the relation between obesity and SRH was similarly strong in all sub-groups, but there was seldom a relation between overweight and SRH. In summary, the results show that obesity is linked to poor SRH regardless of SES and school type, while the relation between overweight and SRH varies by social factors among adolescents.

## 1. Introduction

Self-rated health (SRH) is a construct based on individual perception and assessment of health [1]. It is a commonly used measure in health research [2] that has been found to be a reliable and valid indicator of physical and mental functioning [3,4,5]. SRH has also been shown to be predictive of health care utilization and mortality [6].

Various studies have established that being overweight or obese is a risk factor for poor SRH [7,8,9,10,11]. International studies analyzing the relation between overweight/obesity and SRH show that overweight and especially obese people report poor SRH more often than those of normal weight [12,13,14,15,16,17,18,19]. However, only a few of these studies focus on children and adolescents [20,21,22,23,24]. For Germany, only one study dealing with the relation between overweight/obesity and SRH in this young age group was found [25].

Little attention has been paid to whether the relation between overweight/obesity and SRH varies between different population groups. However, these studies are important for discovering heterogeneities in “pathways to health” [26]. People’s socio-economic status (SES) is an important factor for understanding the health of populations [27]. There is evidence that a social gradient exists in health to the disadvantage of those of low SES [28]. Overweight and obesity are examples that tend to be more frequent in the low SES group [29,30,31]. Several studies show that SES and other social determinants of health affect people’s health directly [32]. Literature indicates that there is also some evidence that SES and other social determinants of health may modify the relation between lifestyles and health [33,34,35], but the evidence is still limited for overweight and obesity. Studies investigating the relation between BMI and SRH show that SES and other social determinants of health do not modify the relation between obesity and SRH. These studies, however, focus on adults [36,37,38]. We found no study that examines the relation between BMI and SRH at a young age taking account of SES or other social determinants of health.

Against that background, the aim of this study was to investigate the relation between overweight/obesity and SRH among adolescents in Germany, and whether or not this relation varies by social factors such as family SES and school type of the adolescents. In particular, it seeks to answer three questions:

1. Is there a relation between overweight/obesity and SRH among adolescents in Germany?
2. If so, does the relation between overweight/obesity and SRH vary by family SES and type of school of the adolescents?
3. Are there gender differences in any relation between overweight/obesity and SRH and in the potential modifying impact of SES and school type?

## 2. Materials and Methods

### 2.1. Study Population

We used data from the German Health Interview and Examination Survey for Children and Adolescents (KiGGS) [39]. Participants were enrolled in two steps. First, 167 study locations (sample points) were chosen. Second, subjects were randomly selected from the official registers of local residents (stratified multistage probability sample). The overall percentage of non-deliverable survey contacts was 5.3%, leaving a target population of n = 26,787. The final study population included 17,641 children and adolescents (8965 boys and 8656 girls), with an overall response rate of 66.6%. Response proportions did not essentially differ by age and gender, but were significantly lower among boys and girls from families with a migration background compared to those from native German families. Besides, response was also significantly lower in larger cities (≥100,000 residents) compared to smaller towns. By analyzing the short non-responder questionnaires, it was proven that the collected data give comprehensive and nationally representative evidence on the health status of children and adolescents aged 0 to 17 years living in Germany. In the KiGGS study, all participants were given a medical and physical examination, and those boys and girls aged 11 and upwards also completed a written questionnaire themselves. The parents of all survey participants filled in a questionnaire and took part in a standardized computer-assisted personal interview. Comprehensive laboratory diagnoses were carried out on blood and urine samples. Details of the KiGGS study design, including the sampling procedure, execution and procedure of the study, data management, quality assurance, and the inclusion of migrants have been published elsewhere, see e.g., [40,41,42,43].

### 2.2. Measures

#### 2.2.1. Overweight and Obesity

We used body mass index (BMI) to assess overweight and obesity in adolescence. This was calculated as the ratio of weight (in kg) to height/length (in m^2^) rounded to three digits. Body height and weight were measured using standard methods by trained staff [44]. Body height was measured without wearing shoes, according to a standardized protocol, to the nearest 0.1 cm using portable devices, and body weight in underwear to the nearest 0.1 kg with a calibrated scale. Overweight and obesity were defined based on gender- and age-specific BMI percentiles of the German reference system developed by Kromeyer-Hauschild *et al.* [45]. According to national guidelines and recommendations from the European Childhood Obesity Group (ECOG) and the Childhood Obesity Working Group of the International Obesity Task Force (IOTF), the 90th and 97th BMI percentile of this reference population are proposed as the respective cut-off points for the definition of overweight and obesity, respectively. The weight class normal weight (10th–90th BMI percentile) was used in this study as the reference group (underweight adolescents were excluded from the statistical analyses).

#### 2.2.2. Self-Rated Health

In the KiGGS study SRH was assessed for adolescents aged 11 to 17 years with a self-completion questionnaire (self-assessment). Parents were also asked for a health assessment of their children (external assessment) [46]. Self-assessment was considered a better indicator to describe SRH than external assessment [23,47], so we used the self-assessment of the adolescents. In KiGGS, the question that was asked in order to determine SRH was phrased according to the World Health Organization (WHO) recommendation [48]: “How would you describe your health in general?”. The five possible answers were: “very good”, “good”, “fair”, “poor”, and “very poor”. For the statistical analyses, we dichotomized these responses into “very good/good” *vs.* “fair/poor/very poor” SRH.

#### 2.2.3. Socio-Economic Status and Type of School

Information on parents’ income, occupational status, and educational and occupational qualifications from the parental questionnaire was used to quantify the SES of the adolescents [46]. Each of the three components was rated with a points system (1‒7 points). The sum was calculated and categorized into the following groups: low SES (3‒8 points), medium SES (9‒14 points), and high SES (15‒21 points). It should be noted that the determined SES groups are statistical constructs, which allow making statements about relatively disadvantaged compared to relatively affluent population groups. In this respect, the low SES group does not necessarily mean “lower class” or “poverty population”. Also belonging to the high SES group is not indicative of the privileges of the upper class or wealth. With regard to type of school, in Germany the school system is applied hierarchically. There are four school types at the secondary level that offer education programs of varying length, depth, and emphasis. The most basic is secondary general school (Hauptschule), followed by advanced intermediate school (Realschule), and most advanced grammar school (Gymnasium) [49]. In the latter type of school, the final exam (Abitur) qualifies for university education. The fourth type of school is comprehensive school (Gesamtschule) that offers options for all three aforementioned school types. For this reason, we considered comprehensive school as a medium school type. For adolescents who had already left school, we used the most recent type of school.

### 2.3. Statistical Analysis

The analyses were based on information from 6813 adolescents aged 11 to 17 years. Of the total sample were 3492 boys and 3321 girls. The average age was 14.4 years (boys: 14.3 years, girls: 14.4 years) (for additional information about the KiGGS sample description, see Table 1). In the first step, we analyzed the relation between BMI and SRH. In the second step, we stratified the analyses by SES and type of school in order to verify whether this relation varies by social factors. In all analyses, SRH was used as the dependent variable and the different BMI groups mentioned previously as independent variables. Both family SES and adolescents’ school type were included in the analyses as modifying variables (stratifying variables). In the third and last step, we performed a sensitivity analysis by using regression analyses with 1-way, 2-way and 3-way interactions of BMI, SES and school type to test how these three variables interact with one another in a single model. BMI, SES and school type were included in these analyses as scale variables. The statistical evaluation was carried out using the statistical software package SPSS 20.0 (SPSS Inc., Chicago, IL, USA). Besides prevalence, the results of binary logistic regressions (odds ratio (OR), 95% confidence intervals (95% CI)) were reported using the SPSS procedure for complex samples that allows for the chosen sampling method in the KiGGS study. Significant differences were reported based on the ORs. To account for the multistage and clustered sample design of KiGGS, a weighting factor was used in the analyses that corrects the deviances of the net sample to the population structure (at 31 December 2004) concerning age (in years), gender, residence (West and East Germany, Berlin), and nationality [40]. To show potential gender differences, all analyses were done for boys and girls separately. Gender differences are shown for the relation between overweight/obesity and SRH and for the potential modifying impact of SES and school type.

**Table 1:** Sociodemographic characteristics of the KiGGS sample (baseline 2003‒2006), participants aged 11 to 17 years (*n* = 6813).

	Total (%)	Boys (%)	Girls (%)
**Age in years: mean (SD)**	14.4 (2.00)	14.3 (1.97)	14.4 (2.03)
**Sex**	6813 (100.0)	3492 (51.3)	3321 (48.7)
**Region of residence**			
Newly formed German states (incl. Berlin)	2278 (33.4)	1148 (32.9)	1130 (34.0)
Old West German states	4535 (66.6)	2344 (67.1)	2191 (66.0)
**Migration background**			
Yes	1054 (15.5)	557 (16.0)	497 (15.0)
No	5755 (84.5)	2934 (84.0)	2821 (84.9)
Missing data	4 (0.1)	1 (0.0)	3 (0.1)
**Self-rated health**			
Very good	1245 (18.3)	730 (20.9)	515 (15.5)
Good	3365 (49.4)	1680 (48.1)	1685 (50.7)
Fair	758 (11.1)	342 (9.8)	416 (12.5)
Poor	29 (0.4)	16 (0.5)	13 (0.4)
Very poor	2 (0.0)	1 (0.0)	1 (0.0)
**BMI classes**			
Underweight	510 (7.5)	281 (8.0)	229 (6.9)
Normal weight	5063 (74.3)	2578 (73.8)	2485 (74.8)
Overweight	692 (10.2)	364 (10.4)	328 (9.9)
Obese	515 (7.6)	254 (7.3)	261 (7.9)
Missing data	33 (0.5)	15 (0.4)	18 (0.5)
**SES**			
Low	1777 (26.1)	916 (26.2)	861 (25.9)
Medium	3192 (46.9)	1625 (46.5)	1567 (47.2)
High	1609 (23.6)	816 (23.4)	793 (23.9)
Missing data	235 (3.4)	135 (3.9)	100 (3.0)
**Type of school**			
Secondary general school	1150 (16.9)	680 (19.5)	470 (14.2)
Intermediate/Comprehensive school	2586 (38.0)	1329 (38.1)	1257 (37.9)
Grammar school	2299 (33.7)	1033 (29.6)	1266 (38.1)
Other school types	550 (8.1)	325 (9.3)	225 (6.8)
Missing data	228 (3.3)	125 (3.6)	103 (3.1)

SD = standard deviation; BMI = body mass index; SES = socio-economic status; Percentages based on weighted data; extrapolated to the residential population of Germany (0‒17 years) on 31 December 2004 (without missing data).

## 3. Results

### 3.1. SRH According to BMI

The prevalence of fair to very poor SRH rose with increasing BMI. This was evident for both boys (normal weight: 11.3%, overweight: 20.1%, obesity: 25.3%) and girls (normal weight: 14.6%, overweight: 19.3%, obesity: 35.7%). Age-adjusted results show that overweight and obese boys and obese girls reported fair to very poor SRH significantly more often than their normal weight peers. Compared with the reference group, overweight boys had a 2.0-fold increased risk and obese boys a 2.7-fold increased risk of fair to very poor SRH (overweight: OR 1.99; 95% CI 1.36‒2.89; obesity: OR 2.68; 95% CI 1.85‒3.89). Obese girls had an increased risk by a factor of 3.2 of fair to very poor SRH compared with normal weight girls (OR 3.23; 95% CI 2.32‒4.49).

Analysis of age-specific differences shows that in early (11‒13 years) and late adolescence (14‒17 years), overweight and obese boys and obese girls were more likely to report fair to very poor SRH than their normal weight peers. Age-adjusted results show that the differences between normal weight and overweight/obese boys in the assessment of fair to very poor SRH were more apparent in early than late adolescence. For girls, this also applied to the relation between obesity and SRH (Table 2).

**Table 2:** Relation between BMI and SRH (fair to very poor) among adolescents aged 11 to 17 years stratified by age (*n* = 4981). Prevalence and age-adjusted odds ratios (OR) with 95% confidence intervals (95% CI).

	Age Groups			
	11‒13 Years		14‒17 Years	
	% (95% CI)	OR (95% CI)	% (95 % CI)	OR (95% CI)
**Boys**				
Normal weight	10.2 (8.0‒12.8)	ref.	11.7 (9.9‒13.6)	ref.
Overweight	20.7 (11.0‒35.5)	2.32 * (1.07‒5.05)	19.8 (14.5‒26.6)	1.89 ** (1.23‒2.91)
Obesity	26.0 (15.7‒40.0)	3.02 *** (1.55‒5.91)	25.1 (18.3‒33.4)	2.56 *** (1.65‒3.97)
**Girls**				
Normal weight	13.2 (10.2‒16.9)	ref.	15.1 (13.0‒17.4)	ref.
Overweight	18.5 (12.1‒27.3)	1.50 (0.84‒2.68)	19.7 (13.7‒27.4)	1.37 (0.87‒2.17)
Obesity	45.2 (32.9‒58.0)	5.42 *** (2.99‒9.84)	32.6 (24.9‒41.4)	2.69 *** (1.76‒4.11)

BMI = body mass index; SRH = self-rated health; ref. = reference group; Significant *p* values compared with normal weight indicated in bold = * *p* < 0.05, ** *p* < 0.01, *** *p* < 0.001.

### 3.2. Impact of SES and Type of School on the Relation between BMI and SRH

Table 3 and Table 4 show the relation between overweight/obesity and SRH stratified by SES and school type. Results show that the prevalence of fair to very poor SRH decreased in all BMI classes from low to high SES and from the lowest to highest school type. The largest group reporting fair to very poor SRH was found among obese boys and girls with low SES and at the lowest school type. The smallest group was found among normal weight boys and girls with high SES and at the highest school type. In all sub-groups, overweight and obese adolescents reported fair to very poor SRH more often than their normal weight peers. Age-adjusted results show that the relation between obesity and SRH was similarly strong in all sub-groups with an approximately 2- to 4-fold increased risk. The only exception was the high SES group and the highest school type for boys. In contrast, the relation between overweight and SRH was observed less often. In boys, this relation was seen in the medium and high SES group and at the middle and highest school type, and in girls solely in the low SES group.

**Table 3:** Relation between BMI and SRH (fair to very poor) among adolescents aged 11 to 17 years stratified by SES (n = 4801). Prevalence and age-adjusted odds ratios (OR) with 95% confidence intervals (95% CI).

	Boys		Girls	
	% (95% CI)	OR (95% CI)	% (95% CI)	OR (95% CI)
**Low SES**				
Normal weight	11.6 (8.9‒15.0)	ref.	17.1 (13.3‒21.7)	ref.
Overweight	18.4 (10.7‒29.8)	1.71 (0.86‒3.40)	30.4 (21.4‒41.2)	2.16 ** (1.21‒3.86)
Obesity	29.3 (18.8‒42.6)	3.14 *** (1.63‒6.06)	40.8 (30.9‒51.5)	3.41 *** (2.00‒5.81)
**Medium SES**				
Normal weight	11.0 (9.0‒13.3)	ref.	16.3 (13.6‒19.3)	ref.
Overweight	18.5 (11.9‒27.7)	1.85 * (1.08‒3.15)	15.1 (9.4‒23.3)	0.92 (0.51‒1.64)
Obesity	22.5 (14.3‒33.6)	2.35 ** (1.30‒4.28)	31.4 (22.3‒42.2)	2.31 ** (1.36‒3.91)
**High SES**				
Normal weight	10.1 (7.3‒13.9)	ref.	8.8 (6.6‒11.7)	ref.
Overweight	22.8 (12.4‒38.3)	2.69 * (1.18‒6.15)	12.0 (5.3‒24.9)	1.46 (0.60‒3.58)
Obesity	19.9 (9.7‒36.6)	2.13 (0.86‒5.27)	25.3 (11.1‒48.1)	3.41 * (1.15‒10.12)

BMI = body mass index; SRH = self-rated health; SES = socio-economic status; ref. = reference group; Significant *p* values compared with normal weight indicated in bold = * *p* < 0.05, ** *p* < 0.01, *** *p* < 0.001.

To check our explorative findings on the impact of BMI in various contexts of parental SES and school type, we calculated logistic regression analyses with 1-way, 2-way and 3-way interactions of BMI, SES and school type. Results show that there was no relation between SRH and SES after controlling for BMI and school type. In addition, the results indicate that regardless of SES, the impact of BMI on SRH was stronger the higher the school type (see Table A1 in the Appendix).

**Table 4:** Relation between BMI and SRH (fair to very poor) among adolescents aged 11 to 17 years stratified by type of school (n = 4831). Prevalence and age-adjusted odds ratios (OR) with 95% confidence intervals (95% CI).

	Boys		Girls	
	% (95% CI)	OR (95% CI)	% (95% CI)	OR (95% CI)
**Secondary general school**				
Normal weight	13.3 (10.1‒17.3)	ref.	22.1 (16.5‒29.0)	ref.
Overweight	21.9 (13.3‒33.6)	1.83 (0.92‒3.64)	29.4 (17.9‒44.3)	1.48 (0.72‒3.06)
Obesity	30.6 (18.6‒45.9)	2.96 ** (1.47‒5.99)	35.9 (24.1‒49.7)	1.98 * (1.05‒3.74)
**Intermediate/Comprehensive school**				
Normal weight	10.6 (8.5‒13.1)	ref.	15.2 (11.2‒18.5)	ref.
Overweight	22.6 (14.6‒33.2)	2.51 ** (1.54‒6.04)	16.5 (10.3‒25.3)	1.11 (0.62‒2.00)
Obesity	30.5 (20.7‒42.5)	3.81 *** (2.17‒6.68)	36.3 (25.9‒48.1)	3.24 *** (1.88‒5.60)
**Grammar school**				
Normal weight	8.5 (6.2‒11.4)	ref.	10.4 (8.3‒13.0)	ref.
Overweight	17.3 (10.2‒27.7)	2.40 * (1.22‒4.78)	16.4 (8.3‒29.8)	1.80 (0.76‒4.23)
Obesity	18.1 (7.0‒39.4)	2.34 (0.77‒7.11)	34.9 (21.4‒51.4)	4.40 *** (2.09‒9.26)

BMI = body mass index; SRH = self-rated health; ref. = reference group. Significant *p* values compared with normal weight indicated in bold = * *p* < 0.05, ** *p* < 0.01, *** *p* < 0.001.

## 4. Discussion

### 4.1. Results of the German KiGGS Study

The results of the KiGGS study show that overweight boys and obese boys and girls reported fair to very poor SRH more often than normal weight peers. Age-differentiated analysis demonstrates that the differences between normal weight and overweight/obese boys were more apparent in early (11‒13 years) than late adolescence (14‒17 years). For girls, this also applied to the relation between obesity and SRH. Obese girls in early adolescence were the main risk group for fair to very poor SRH.

Particular attention was paid to whether the relation between overweight/obesity and SRH varies by SES and school type. In all sub-groups, overweight and obese boys and girls reported fair to very poor SRH more often than their normal weight peers. Age-adjusted results show that the relation between obesity and SRH was similarly strong in all sub-groups (OR between 2.0 and 4.4) and therefore did not vary by SES and school type. However, the relation between overweight and SRH varied by SES and school type. In boys, the relation between overweight and SRH was shown in those with medium and high SES and at the middle and highest school type. In girls, this relation was seen only in those with low SES (in girls, there was no relation between overweight and SRH at any school type).

### 4.2. KiGGS Data in the Light of Previous Research

The KiGGS results on the relation between overweight/obesity and SRH are in line with previously published international studies [20,21,22,23,24]. However, it must be noted that SRH is often dichotomized differently. For example, Mota *et al.* [24] investigated the relation between overweight/obesity and SRH for Portuguese girls aged 10 to 18 years. Logistic regression analyses showed that obese girls reported negative SRH (“fair/poor” *vs.* “excellent/very good/good”) more often than normal weight girls. Using data from two nationally representative surveys in the United States, Skinner *et al.* [21] indicated that overweight children and adolescents aged 6 to 17 years reported excellent SRH (“excellent” *vs.* “very good/good/fair/poor”) less often than their healthy weight peers. A Canadian study examined the relation between obesity and SRH among boys and girls aged 8 to 10 years by using logistic regressions. Herman *et al.* [22] showed that the ORs, which reported less than excellent SRH (“excellent” *vs.* “very good/good/fair/poor” SRH), were higher among obese boys and girls compared with normal weight peers. For Germany, only one regional study from Bavaria that focused on the relation between obesity and SRH among 12- to 24-year-olds could be found. Using logistic regression models, Schulz *et al.* [25] showed that obese boys and girls had a greater risk for poor SRH (“poor/less good/satisfying” *vs.* “very good”) than their normal weight peers.

While many of the aforementioned studies of the relation between BMI and SRH stratified the analyses by gender, no study has currently stratified the analyses by age, SES and school type to examine whether the relation between overweight/obesity and SRH varies by age and social factors among adolescents. Our results show that obese boys and girls reported fair to very poor SRH more often in early than late adolescence. In this context, it should be noted that early and late adolescence are different in terms of cognitive and social functioning [50,51]. Studies suggest that, especially in early adolescence, obese boys and girls have lower self-esteem and mental health issues such as behavioral problems and depressive symptoms [52,53,54]. A few studies have focused on the relation between obesity, SRH and social factors among adults [36,37,38]. Laaksonen *et al.* [37] found that SES did not modify this relation. Mansson and Merlo showed that SRH was related to obesity and SES, but no evidence of interaction between them leading to a synergistic effect on SRH was found [38]. In contrast, Bethea *et al.* [36] showed that regarding obesity, educational attainment and household income were significant contributors to SRH.

### 4.3. Strengths and Limitations

The KiGGS study has many advantages, the most important one being that it provides for Germany nationally representative data of different health indicators, such as overweight and obesity, over the entire age range of children and adolescents. Another advantage is that body height and weight were measured instead of using self-reported data. It is well known that self-reports undervalue the prevalence of overweight and obesity because people tend to underreport their BMI [55,56]. Despite these strengths, some limitations need to be discussed. Most importantly, our analyses are based on a cross-sectional survey, which is why no definite statement on causality or causal direction can be made. Whether poor SRH is cause or consequence of overweight and obesity cannot be answered with these data. In future, KiGGS will become a cohort study [57], which may contribute to a better understanding of the cause-effect relations between overweight/obesity and SRH. The first telephone follow-up survey was completed in 2012, and from September 2014 a new health survey with a medical section is currently being conducted. Once this survey has been completed, survey and measurement data will be available over a 10-year period. Second, we chose common, but arbitrary cut-off values for SRH. Therefore, we performed a sensitivity analysis by using ordered logistic regression models to test whether those had an effect on our interpretation of the data. Overall, these results confirmed our original results. Additionally, we tested whether the loss of efficiency due to the highly stratified analyses had an effect on the results using fully specified logistic models with 3-way interactions of sex, BMI, age and SES/school type. These tests also confirmed our original estimates.

## 5. Conclusions

The KiGGS results show that overweight boys and, in particular, obese boys and girls report fair to very poor SRH more often than their normal weight peers. The effects of overweight and obesity on children`s health are currently the subject of considerable research. Numerous studies have shown that overweight and obesity affect the health of young people in many areas. Overweight and obese adolescents have, for example, a reduced health-related quality of life [58,59,60], more mental health problems [61,62,63], and lower self-esteem [64,65,66] than those of normal weight. Based on the analyses stratified by social factors, we analyzed whether the effects of overweight and obesity on SRH vary between different population groups. The results show that the effect of overweight on SRH is likely to vary by SES and school type, namely in boys in favor of the low SES group and in girls in favor of the high SES group. In contrast to overweight, obesity is linked to poor SRH regardless of SES and school type, and therefore the effect of obesity on SRH does not vary by social factors. There has been and still is a call for interventions to combat overweight and obesity focusing on boys and girls with low SES [32]. It has been shown, however, that low SES is a barrier to interventions against overweight and obesity because those who benefit most are almost exclusively children and adolescents with high SES [67]. In Britain, the Department of Health published in 2010 a White Paper setting out a “radical new approach” to improve the health and well-being of the British population and to reduce social inequalities. Given the fact that some population groups face social barriers and need more support, it is recommended in the White Paper to develop tailor-made approaches for different population groups (such as different SES or education groups) taking account of the existing social barriers [68]. Our findings suggest that this approach is appropriate and should be used more widely.

## Appendix

**Table A1:** Results of the multivariable logistic regression models with SRH as dependent variable, adjusted for sex and age.

	Model 1 ^a^		Model 2 ^b^		Model 3 ^c^	
	OR (95% CI)	*p* value	OR (95% CI)	*p* value	OR (95% CI)	*p* value
**Sex (boys)**	0.7 (0.6–0.8)	0.000	0.7 (0.6–0.8)	0.000	0.7 (0.6–0.8)	0.000
**Sex (girls)**	–	–	–	–	–	–
**Age**	1.0 (0.9–1.0)	0.264	1.0 (0.9–1.0)	0.236	1.0 (0.9–1.0)	0.241
**BMI**	1.1 (1.1–1.1)	0.000	1.1 (1.0–1.2)	0.057	1.1 (1.0–1.1)	0.142
**SES**	1.0 (1.0–1.0)	0.241	1.1 (1.0–1.3)	0.142	1.1 (1.0–1.3)	0.119
**School type**	0.8 (0.7–0.9)	0.000	0.4 (0.2–0.7)	0.003	0.4 (0.2–0.7)	0.005
**BMI/SES**	–	–	1.0 (1.0–1.0)	0.085	1.0 (1.0–1.0)	0.317
**BMI/School type**	–	–	1.0 (1.0–1.1)	0.022	1.0 (1.0–1.1)	0.014
**BMI/SES/School type**	–	–	–	–	1.0 (1.0–1.0)	0.235

OR = odds ratios; 95% CI = 95% confidence intervals; *p* = *p*-value BMI= body mass index; SRH = self-rated health; SES = socio-economic status; **^a^** 1-way interaction of BMI, SES and school type; **^b^** 2-way interaction of BMI, SES and school type; **^c^** 3-way interaction of BMI, SES and school type.
